# Bayesian spatio-temporal modeling of childhood anemia in Tanzania: spatial distribution, determinants, and causal impact of malaria

**DOI:** 10.3389/fpubh.2026.1919182

**Published:** 2026-10-21

**Authors:** Lembris Laanyuni Njotto, Wilfred Senyoni, Angelina Mageni Lutambi

**Affiliations:** 1 Department of Mathematics and ICT, College of Business Education, Dar Es Salaam, Tanzania; 2 College of Information and Communication Technologies, University of Dar Es Salaam (CoICT - UDSM), Dar Es Salaam, Tanzania; 3 Dodoma Research Centre, National Institute for Medical Research, Dodoma, Tanzania

**Keywords:** anemia, attributable fraction, Bayesian spatio-temporal, causal effect, children under 5 years, number needed to treat

## Abstract

**Introduction:**

Childhood anemia remains a major public health challenge in low- and middle income countries, impairing growth, cognition, and child survival. In Tanzania, the burden remains persistently high, yet its spatial patterns, temporal trends, and links to infectious diseases, particularly malaria, are not well understood.

**Methods:**

Using four rounds of the Tanzania Demographic and Health Survey (2007/08–2022) and a Bayesian spatio-temporal geostatistical framework with survey-weighted causal inference, we mapped anemia prevalence and quantified the impact of malaria through estimates of average causal effects (ACE), attributable fractions (AF), and numbers needed to treat (NNT).

**Results:**

Anemia prevalence remained high (60.1% overall), with persistent clusters in the western, southern, and Lake Zone areas; prevalence declined from 2007/08 to 2015/16 before rising again in 2022. Malaria emerged as the strongest predictor of anemia, and its effect strengthened with child age: the odds ratio increased from 2.89 (95% CrI: 2.25–3.72) among children aged 6–23 months to 4.07 (3.53–4.71) among those aged 42–59 months. The ACE of malaria peaked at 0.266 in 2015/16, with an AF of 6.4% and the lowest NNT (3.93) recorded in the same year, indicating malaria prevention was most efficient during this period. Minimum temperature above approximately 18°C was associated with increased anemia risk, while precipitation showed no credible association once malaria status was already accounted for; anemia risk was lower at mid-range cluster altitudes (~1,000–1,600 m), consistent with reduced malaria transmission at these elevations. Lower maternal education, household poverty, larger household size, and younger maternal age were also associated with higher anemia risk.

**Conclusion:**

These findings indicate that childhood anemia in Tanzania is driven by entrenched spatial heterogeneity and a strong, age-dependent, time-varying causal relationship with malaria, highlighting the need for geographically targeted, multisectoral strategies that integrate malaria control with broader social, economic, and environmental interventions.

## 1 Introduction

Childhood anemia remains one of the most persistent and pressing global public health challenges, affecting growth, cognitive development, and survival among millions of children under 5 years of age (1, 2). The condition, defined by low levels of hemoglobin or red blood cells, limits oxygen delivery to tissues and the developing brain, impairing both physiological and cognitive functions (3). Despite global health initiatives and nutritional interventions, anemia continues to affect nearly 40% of children under 5 years of age and one-third of women of reproductive age, with the heaviest burden concentrated in sub-Saharan Africa and South Asia (1).

The etiology of childhood anemia is multifactorial, encompassing nutritional, infectious, and socioeconomic determinants. Iron deficiency remains the leading cause, often driven by inadequate dietary intake or poor nutrient absorption (2). However, in many tropical regions, infectious diseases, notably malaria, HIV, and parasitic infections, amplify the burden by destroying red blood cells and disrupting iron metabolism (4). These biomedical causes are further compounded by contextual factors such as poverty, food insecurity, poor sanitation, and limited access to healthcare, forming a persistent cycle of infection, undernutrition, and socioeconomic vulnerability (5). In Tanzania, childhood anemia has persisted as a major public health concern despite continuous national and international interventions. Data from the Tanzania Demographic and Health Survey and Malaria Indicator Survey rounds indicates a persist high prevalence among children aged 6–59 months: 68.8% in 2007/08, 56.5% in 2011/12, 57.4% in 2015/16, and 59.7% in 2022 (6–9), withe the highest rates consistently occurring among children under 2 years of age.

Tanzania has implemented several multisectoral strategies, such as the National Multisectoral Nutrition Action Plan (NMNAP) (10) and national malaria control programs (11), to address both nutritional and infectious causes of anemia. Nonetheless, the limited decline in prevalence suggests that existing interventions may not adequately capture or respond to the complex and localized drivers of the condition. Anemia distribution is highly heterogeneous. Regions with intense malaria transmission (12), poor dietary diversity, and restricted access to healthcare consistently exhibit higher prevalence. Yet much of the available evidence relies on national averages, which obscure local disparities and fail to capture temporal dynamics. Conventional statistical methods often overlook spatial dependence and time varying relationships, reducing their capacity to identify high risk areas or disentangle the interacting effects of socioeconomic, environmental, and infectious determinants. This highlights a critical need for integrated spatio-temporal approaches capable of revealing fine scale variations in anemia risk and quantifying the causal relationship between malaria and anemia, an understanding that is vital for designing geographically targeted, evidence based interventions.

This study used a Bayesian spatio-temporal geostatistical modeling framework to investigate the distribution and determinants of childhood anemia in Tanzania. The model captured spatial and temporal patterns of anemia among children under 5 years of age, accounting for demographic, socioeconomic, and environmental factors, and further generated predictions of anemia prevalence in unsampled locations. In addition to the spatio-temporal analysis, the study quantified the causal impact of malaria on childhood anemia by estimating the average causal effect (ACE), the attributable fraction (AF), and the number needed to treat (NNT). This causal analysis provided an understanding of the contribution of malaria to the burden of anemia in children.

## 2 Materials and methods

### 2.1 Data

This study utilized data from the Tanzania Demographic and Health Surveys (TDHS) conducted in 2007/08, 2011/12, 2015/16, and 2022, which were obtained from the DHS program website (13). The DHS offers nationally representative statistics on a broad spectrum of population, health, and nutrition indicators, including anemia and malaria among children under 5 years of age. These surveys employ a stratified, two-stage cluster sampling method to ensure both national and regional representativeness. In the initial stage, enumeration areas (EAs) are selected from the most recent national census framework, stratified by region and classified as urban or rural. The second stage involves systematically selecting a predetermined number of households from updated household listings within each EA, thereby forming sampling clusters, which act as the smallest geographical units for sampling purposes.

The geographical coordinates (GPS) of each sampling cluster is recorded to enable both spatial and temporal analyses. Within each selected household, women were interviewed using a structured questionnaire, which collects detailed information on demographic, socioeconomic, and health-related characteristics. Children under 5 years of age, who are present in the household on the survey day, including visitors, are tested for anemia and malaria. Whilst anemia and malaria test results are measured directly from the children, pertinent maternal and household characteristics including mother's age, level of education, and household wealth index, are derived from the mother of the child and from the household records. Each child was linked to her own birth-history record in the Children's Recode using the cluster, household, and household-roster line-number identifiers, with agreement between the linked record's child age and sex and the corresponding fields in the child's own record verified for every match. This linkage succeeded for 86.8% of children; the remaining 13.2% were not living with an identifiable biological mother at the time of interview (e.g., grandparent- or other relative-led care). For these children, a separate “no identifiable resident mother” indicator was retained as a covariate rather than discarding the child from the analysis. This linkage allows for a comprehensive assessment of child health outcomes in relation to maternal, household, and broader contextual factors. The sample sizes for each DHS survey included in this study, including the number of children surveyed, the total sampled clusters, and the average number of children per cluster, are summarized in Table 1. Notably, the average number of children per cluster differed across surveys, with figures ranging from approximately 15.09 in 2015/16 to 8.02 in 2022.

**Table 1:** Summary of sample sizes from the analyzed DHS data, including the number of surveyed children, number of sampled clusters, and the average number of children per cluster.

Survey year	No. of clusters	No. of children	Average children per cluster
2007/08	474	6,387	13.47
2011/12	583	7,606	13.05
2015/16	607	9,161	15.09
2022	623	4,997	8.02

Anemia status was defined using the standard DHS biomarker protocol: capillary blood hemoglobin concentration was measured in the field using a battery-operated HemoCue photometer, adjusted for the cluster's altitude following the standard DHS/CDC correction, and a child was classified as anemic if adjusted hemoglobin was below 11.0 g/dL, the WHO threshold for children aged 6–59 months (14). Malaria status was determined using a rapid diagnostic test (RDT) performed in the field at the time of the interview, using the same finger-prick or heel-prick blood sample collected for anemia testing. Climatic data were obtained from the WorldClim global climate database (15, 16), which provides high-resolution gridded climate surfaces derived from interpolated weather station data. Monthly temperature and precipitation related variables were extracted and spatially matched to the study locations and temporally aligned with the survey years and months to generate relevant climate exposure variables for the analysis.

### 2.2 Spatio-temporal geostatistical model

To estimate anemia prevalence at unsampled locations and evaluate the influence of covariates, we adopted a Bayesian spatio-temporal geostatistical modeling approach (17). Prior to model fitting, all cluster GPS coordinates were projected from geographic coordinates (WGS84) to the Universal Transverse Mercator Zone 37S projection (EPSG:32737), providing planar distances in meters that are required by the SPDE triangulation mesh. To confirm the presence of spatial autocorrelation in anemia prevalence before specifying a spatial model, we conducted Moran's *I* test using region-level average anemia prevalence and queen-contiguity spatial weights across the 31 administrative regions of Tanzania. The test yielded a Moran's *I* statistic of 0.495 (*p* < 0.0001), indicating positive spatial clustering at the regional level. Since this test was performed on aggregated regional averages rather than at the cluster level at which the spatial random field is specified, we treat it as a heuristic indication of spatial dependence rather than a formal test of cluster-level autocorrelation; the contribution of the spatial field at the cluster level is instead reflected in the estimated spatial range and variance of the fitted model.

Let $S\subset {\mathbb{R}}^{2}$ denote the spatial domain of interest. Children are sampled within survey clusters indexed by *i* = 1, …, *S*. For each child *j* observed in cluster *i* at survey time *t*∈{2007/08, 2011/12, 2015/16, 2022}, let *Y*_*ijt*_∈{0, 1} denote the binary anemia status (Equation 1). Each cluster has projected coordinates $s_{it}=\left({\text{easting}}_{it},{\text{northing}}_{it}\right)\in S$ (UTM Zone 37S, meters), and multiple children may be observed at the same cluster location. We assume that the response variable follows a Bernoulli distribution:


$$\begin{array}{c} Y_{ijt}\sim \text{Bernoulli}\left(p_{ijt}\right), \end{array}$$ (1)

where *p*_*ijt*_ denotes the probability that child *j* at location *s*_*it*_ and time *t* is anemic. To model the relationship between *p*_*ijt*_ and relevant covariates (Equation 2), we adopt a logistic framework:


$$\begin{array}{l} \text{logit}\left(p_{ijt}\right)=\eta_{ijt},\\ \;\eta_{ijt}=\beta_{0}+\overset{K}{\underset{k=1}{\sum }}\beta_{k}X_{ijt}^{\left(k\right)}+\overset{H}{\underset{h=1}{\sum }}f_{h}\left(Z_{h,ijt}\right)+u_{c\left(i\right)}+v_{s\left(i\right)}+\xi \left(s_{it},t\right), \end{array}$$ (2))

where β_0_ is the intercept and β_*k*_ are fixed-effect coefficients associated with the covariates $X_{ijt}^{\left(k\right)}$, including child-level, maternal, and household characteristics. The terms *u*_*c*(*i*)_ and *v*_*s*(*i*)_ denote independent and identically distributed random effects for survey clusters and sampling strata, respectively, accounting for unobserved heterogeneity arising from the survey design. The functions *f*_*h*_(·) represent smooth, non-linear effects of continuous covariates, including climatic variables, child age, maternal age, altitude, and survey year. To ensure computational tractability with over 27,000 observations, continuous climate covariates were first discretised into 25 equal-frequency bins using the inla.group() function (18), following standard practice for second-order random walk smooths in large datasets. These smooth terms were modeled using random walk priors, which incorporate information from the preceding value(s) in the sequence, enabling the model to capture non-linear trends without imposing restrictive parametric assumptions (19). Both first-order random walk (RW1) and second-order random walk (RW2) were evaluated during model development. RW1 assumes a smoothly evolving effect across the covariate's range and penalizes abrupt changes between adjacent values, making it appropriate when the underlying relationship is expected to be simple. RW2 extends this by also penalizing changes in the slope, allowing the fitted effect to curve, which makes it more suitable when the covariate's relationship with the outcome is expected to be non-linear. Each covariate was tested under both prior structures during model development, and the best-supported prior for each term was retained. Finally, ξ(*s*_*it*_, *t*) is a spatio-temporal random effect capturing unobserved spatial and temporal heterogeneity shared across children at the same location and time.

The latent process ξ(*s*_*it*_, *t*) evolves over time according to a first-order autoregressive (AR(1)) model (17, 20):


$$\xi \left(s_{it},t\right)=a\xi \left(s_{it},t-1\right)+\varphi \left(s_{it},t\right),$$

where |*a*| < 1 governs the strength of temporal dependence, and φ(*s*_*it*_, *t*) is a spatially correlated innovation term. The initial state is assumed to follow a stationary distribution:


$$\xi \left(s_{it},1\right)\sim N\left(0,\frac{\sigma_{\varphi }^{2}}{1-a^{2}}\right).$$

The innovation term φ(*s*_*it*_, *t*) is modeled as a zero-mean Gaussian process that is temporally independent but spatially correlated (20). Its spatio-temporal covariance structure is defined as:


$$\text{Cov}(\varphi \left(s_{i},t\right),\varphi \left(s_{\ell },u\right))=\{\begin{array}{ll} 0, & \text{if }t\neq u,\\ C_{s}\left(s_{i},s_{\ell }\right), & \text{if }t=u, \end{array}$$

where *C*_*s*_(*s*_*i*_, *s*_ℓ_) is the spatial covariance function, that can be modeled using a Gaussian random field (GRF) with Matérn covariance function defined as:


$$C_{s}\left(s_{i},s_{\ell }\right)=\frac{\sigma_{\varphi }^{2}}{2^{\nu -1}\text{Γ}\left(\nu \right)}{\left(\kappa ||s_{i}-s_{\ell }||\right)}^{\nu }K_{\nu }\left(\kappa ||s_{i}-s_{\ell }||\right),$$

with ||*s*_*i*_−*s*_ℓ_|| denoting the Euclidean distance between spatial locations *s*_*i*_ and *s*_ℓ_, $\sigma_{\varphi }^{2}$ the marginal variance of the spatial field, and *K*_ν_ the modified Bessel function of second kind and order ν>0. The integer value ν controls the smoothness of the field and is typically fixed for identifiability. The spatial scaling parameter κ>0 is related to the range ρ, which represents the distance at which the correlation between two points becomes negligible. Specifically $\rho =\frac{\sqrt{8\nu }}{\kappa }$, and at this distance the spatial correlation is close to 0.1 (20, 21).

We fitted a spatio-temporal geostatistical model using the Integrated Nested Laplace Approximation (INLA), implemented via the R-INLA package in R (version 4.6.1) (18, 22). INLA offers a computationally efficient alternative to Markov Chain Monte Carlo (MCMC) methods and is specifically designed for approximate Bayesian inference in latent Gaussian models (23, 24). It combines analytical approximations with numerical integration to derive posterior distributions of model parameters, which can then be post-processed to compute quantities of interest such as posterior means, variances, and quantiles. The spatial component of the latent field, denoted by *C*_*s*_, was approximated using a Gaussian Markov Random Field (GMRF) representation through the Stochastic Partial Differential Equation (SPDE) approach (24). The triangulated mesh was constructed over the Tanzania domain with a minimum triangle edge length of 50 km and a maximum edge length of 100 km in the interior and 200 km in the outer extension.

Penalized complexity (PC) priors (25) were specified for the SPDE hyperparameters: *P*(ρ < 300km) = 0.05 for the spatial range, reflecting a prior belief that correlation extends beyond 300 km, and *P*(σ>1) = 0.05 for the marginal standard deviation on the logit scale. For the AR(1) temporal autocorrelation parameter *a*, a PC prior with *P*(*a*>0) = 0.9 was used, reflecting an expectation of positive temporal persistence. Due to the large model size (701 mesh nodes × 4 survey periods, yielding 2,804 latent spatial dimensions, combined with RW1/RW2 smooth terms and two iid random effects fitted on more than 27,000 observations), the empirical Bayes integration strategy (int.strategy = ~eb~) was adopted for the primary model fit, fixing hyperparameters at their posterior mode. This strategy is recommended by the INLA developers for spatio-temporal SPDE models of this scale (23, 26). To account for unequal selection probabilities arising from the stratified cluster sampling design, DHS survey weights were applied by scaling each observation's likelihood contribution proportionally to its design weight, in accordance with the *DHS Guide to Statistics* (27).

### 2.3 Causality analysis

A major challenge in drawing causal inferences from cross-sectional studies is the issue of reverse causality, where it can be difficult to determine the true direction of the relationship between variables. In the context of malaria and anemia, biological research clearly demonstrates that malaria leads to the development of anemia (28), and there is no evidence to suggest that anemia increases a person's susceptibility to malaria. This biological argument, does not by itself establish temporality in our data: because malaria status and hemoglobin are measured at the same cross-sectional visit, the data alone cannot verify that malaria infection preceded the anemia episode being measured, and we rely on external evidence, not the DHS data, to support this ordering. We treat this as an unresolved limitation of the causal analysis. Despite this clarity in biological causality, incorporating spatial effects into the causal estimation process was not feasible due to the heavy computational demands of integrating over latent spatial structures. While broader geostatistical models can account for spatial heterogeneity, including these effects in counterfactual or causal analyses would require complex integration methods that are beyond this study. To effectively quantify the causal impact of malaria on anemia, we therefore focus on three established epidemiological measures: the Attributable Fraction (AF) (29, 30), the Number Needed to Treat (NNT) (30, 31), and the Average Causal Effect (ACE) (32). All three quantities refer to the same hypothetical population-level contrast: a comparison between the observed distribution of malaria exposure and a counterfactual scenario in which malaria exposure is removed prospectively, before it could affect hemoglobin, analogous to a fully effective malaria-prevention programme (e.g., vector control or chemoprevention) rather than treatment of an infection already in progress. This distinction matters most for the NNT, where a treatment framing would be materially misleading.

The AF quantifies the proportion of anemia cases that would be prevented if malaria were hypothetically prevented across the population (33). That is


$$\begin{array}{c} AF=1-\frac{P\left(Y_{0}=1\right)}{P\left(Y=1\right)}, \end{array}$$ (3)

where *P*(*Y* = 1) is the observed anemia prevalence (factual probability), and *P*(*Y*_0_ = 1) is the counterfactual probability of anemia under the population-level prevention scenario, both standardized over the covariate distribution of the entire study population.

The NNT is conventionally used to summarize the per-person benefit of a preventive intervention. Here, we apply it strictly in the preventive sense: it represents the average number of currently malaria-positive children who would have needed to be prevented from acquiring malaria prior to infection in order to avert one additional case of anemia (30). This does not refer to treating children after infection, as treatment cannot retroactively reverse anemia that has already developed as a consequence of the malaria episode. Throughout this paper, *T*_*m*_ = 1 denotes malaria-positive status and *T*_*m*_ = 0 denotes malaria-negative status. The NNT is defined as:


$$\begin{array}{l} NNT=\\ \frac{1}{{𝔼}_{X|T_{m}=1}\left[p\left(Y=1|T_{m}=1,X\right)\right]-{𝔼}_{X|T_{m}=1}\left[p\left(Y=1|T_{m}=0,X\right)\right]}, \end{array}$$ (4)

where *p*(*Y* = 1∣*T*_*m*_ = 1, *X*) denotes the factual probability of anemia among currently malaria-positive children, conditional on covariates *X*, and *p*(*Y* = 1∣*T*_*m*_ = 0, *X*) denotes the counterfactual probability of anemia for those currently malaria-positive children had their malaria been prevented. Both expectations *𝔼*_*X*∣_*T*__*m*_ = 1_[·] are taken over the covariate distribution of the malaria-positive subgroup specifically, representing the per-person efficiency of malaria prevention for children currently at risk.

ACE indicates the anticipated difference in the likelihood of anemia among children under 5 years of age, comparing a scenario if all are exposed to malaria with one in which none are exposed, under the population-level prevention scenario. This measure captures the impact of malaria exposure on anemia at the population level, standardized over the covariate distribution of the entire study population (in contrast to the NNT, which is restricted to the malaria-positive subgroup). ACE can be expressed in terms of counterfactual and factual probabilities as:


$$\begin{array}{c} ACE={𝔼}_{X}\left[p\left(Y=1|T_{m}=1,X\right)\right]-{𝔼}_{X}\left[p\left(Y=1|T_{m}=0,X\right)\right], \end{array}$$ (5)

where *p*(*Y* = 1∣*T*_*m*_ = 1, *X*) denotes the factual probability of anemia among children exposed to malaria, conditional on covariates X, *p*(*Y* = 1∣*T*_*m*_ = 0, *X*) denotes the counterfactual probability of anemia for each child in the population had they not been exposed, and *E*_*X*_[·] indicates that the expectation is taken over the distribution of covariates X in the entire study population.

To facilitate the estimation of factual and counterfactual probabilities, causal quantities were estimated from separate, year-stratified logistic regression models rather than from the spatio-temporal INLA model. This was necessary because integrating over the latent spatial field during counterfactual prediction is computationally intractable for models of this scale. Each year-specific model regressed childhood anemia on malaria status and the same set of socioeconomic, demographic, and environmental covariates included in the spatio-temporal model. Identifying these quantities causally requires the following assumptions: (i) *conditional exchangeability*, {*Y*(1), *Y*(0)}⊥*T*_*m*_∣*X* — that is, within strata defined by the observed covariates *X*, malaria status behaves as if randomly assigned with respect to the potential anemia outcomes, not merely the weaker claim that there are simply “no unmeasured confounders.” This is a strong assumption, as several plausible shared causes of malaria and anemia (nutritional status, sickle cell trait, recent antimalarial use, and others) are not observed in *X*. (ii) *Conditional positivity*, 0 < *P*(*T*_*m*_ = 1∣*X*) < 1 for every value of *X* observed in the data — every covariate stratum must contain both malaria-positive and malaria-negative children; we did not identify structurally empty strata, though this was not formally tested. (iii) *Consistency*, *Y* = *Y*(*T*_*m*_)—each child's observed anemia outcome under their observed malaria status equals the potential outcome under that same status, which further presumes no meaningfully different “versions” of malaria exposure (e.g., by *Plasmodium* species or parasite density) that would produce materially different anemia risk. (iv) *Temporality*, as mentioned earlier, this is not verifiable from these cross-sectional data. The ACE (Equation 5) was estimated via G-computation (standardization): the fitted logistic model was applied twice to the full dataset, first setting malaria exposure to 1 for every child (*T*_*m*_ = 1, all exposed) and then setting it to 0 for every child (*T*_*m*_ = 0, all unexposed), while holding all other covariates at their observed values. The individual predicted probabilities were averaged over the empirical covariate distribution in each step, and the ACE was computed as the difference between these two marginal means. Point estimates and predictions were weighted by the DHS sample weight to account for the survey's unequal selection probabilities, and 95% confidence intervals were obtained from a stratified cluster bootstrap to account for the clustered, stratified survey design. Standardization followed the logic implemented by the stdGlm() function in the stdReg package (30) in R (version 4.6.1) (22), adapted to incorporate survey weights and the cluster-bootstrap variance.

## 3 Results

### 3.1 Description of the study population

A total of 28,151 children aged 6–59 months were sampled across four nationally representative survey rounds: 6,387 in 2007/08, 7,606 in 2011/12, 9,161 in 2015/16, and 4,997 in 2022. Of these, 269 children (0.96%) were in clusters with no GPS coordinates and were excluded from every model that requires a spatial location or altitude, leaving 27, 882 children for the year-stratified causal models. The mean age of children across all surveys was 32.13 months (SD = 15.56; Table 2). The prevalence of anemia remained high throughout the study period, with 60.1% of children classified as anemic. Boys constituted just over half of the sample (50.3%), and the majority of children (80.5%) resided in rural areas. Malaria positivity was observed in 10.8% of children, with the highest prevalence recorded in 2007/08 (13.2%) and the lowest in 2022 (6.9%).

**Table 2:** Socio-economic, demographic, environmental, and climatic characteristics of children under 5 years of age in Tanzania by survey year and overall.

Status	Variables							Survey year								Overall					
								2007/08		2011/12		2015/16		2022		Total (*N*)			Percent (%)		
								*N*	%	*N*	%	*N*	%	*N*	%						
Anemia status	Negative							1,995	31.24	3,310	43.52	3,901	42.58	2,016	40.34	11,222			38.86		
	Positive							4,392	68.76	4,296	56.48	5,260	57.42	2,981	59.66	16,929			60.14		
Malaria status	Negative							5,546	86.83	6,855	90.13	8,059	87.97	4,653	93.12	25,113			89.21		
	Positive							841	13.17	751	9.87	1,102	12.03	344	6.88	3,038			10.79		
Child gender	Female							3,174	49.69	3,765	49.50	4,589	50.09	2,477	49.57	14,005			49.75		
	Male							3,213	50.31	3,841	50.50	4,572	49.91	2,520	50.43	14,146			50.25		
Place of residence	Rural							5,370	84.08	6,511	85.60	7,120	77.72	3,670	73.44	22,671			80.53		
	Urban							1,017	15.92	1,095	14.40	2,041	22.28	1,327	26.56	5,480			19.47		
Slept under net	No							4,304	67.39	2,293	30.15	4,069	44.42	1,779	35.60	12,445			44.21		
	Yes							2,083	32.61	5,313	69.85	5,092	55.58	3,218	64.40	15,706			55.79		
Mother's highest education	None							1,584	24.80	1,640	21.56	1,760	19.21	925	18.51	5,909			20.99		
	Primary							3,486	54.58	4,263	56.05	4,784	52.22	2,271	45.45	14,804			52.59		
	Secondary school or above							513	8.03	709	9.32	1,416	15.46	10,75	21.51	3,713			13.19		
	No identifiable resident mother							804	12.59	994	13.07	1,201	13.11	726	14.53	3,725			13.23		
Number of household members	1–5							2,145	33.58	2,411	31.70	3,275	35.75	2,086	41.75	9,917			35.23		
	6–10							3,364	52.67	3,880	51.01	4,513	49.26	2,368	47.39	14,125			50.18		
	11 and more							878	13.75	1,315	17.29	1,373	14.99	543	10.87	4,109			14.60		
Family wealth quintile	Poorest							1,308	20.48	1,669	21.94	2,098	22.90	1,069	21.39	6,144			21.83		
	Poorer							1,271	19.90	1,679	22.07	1,907	20.82	992	19.85	5,849			20.78		
	Middle							1,316	20.60	1,657	21.79	1,818	19.84	1,052	21.05	5,843			20.76		
	Richer							1,431	22.40	1,518	19.96	1,860	20.30	1,007	20.15	5,816			20.66		
	Richest							1,061	16.61	1,083	14.24	1,478	16.13	877	17.55	4,499			15.98		
Toilet facility	Unimproved							5,875	91.98	5,355	70.40	2,878	31.42	1,455	29.12	15,563			55.28		
	Improved							512	8.02	2,251	29.60	6,283	68.58	3,542	70.88	12,588			44.72		
Water source	Unimproved							3,714	58.15	5,079	66.78	6,868	74.97	4,039	80.83	19,700			69.98		
	Improved							2,567	40.19	2,486	32.68	2,293	25.03	958	19.17	8,304			29.50		
	Missing/not recorded							106	1.66	41	0.54	0	0.00	0	0.00	147			0.52		
Wall exterior main material	Grass/mud/wood							2,479	38.81	84	1.10	35	0.38	140	2.80	2,738			9.73		
	Bricks/Blocks/Stone							3,855	60.36	5,409	71.11	7,056	77.02	3,201	64.06	19,521			69.34		
	Missing/not recorded							53	0.83	2,113	27.78	2,070	22.60	1,656	33.14	5,892			20.93		
Roof main material	Grass/thatch/mud							3,103	48.58	3,021	39.72	6	0.07	141	2.82	6,271			22.28		
	Iron sheets/tiles/concrete/asbestos							3,214	50.32	0	0.00	330	3.60	121	2.42	3,665			13.02		
	Missing/not recorded							70	1.10	4,585	60.28	8,825	96.33	4,735	94.76	18,215			64.70		
Continuous variables				2007/08				2011/12				2015/16				2022			
				Min.	Mean	SD	Max.	Min.	Mean	SD	Max.	Min.	Mean	SD	Max.	Min.	Mean	SD	Max.
Age of child (months)				6	32.16	15.67	59	6	32.14	15.48	59	6	31.94	15.61	59	6	32.45	15.44	59
Maternal age (years)				15	29.64	6.57	49	15	29.74	6.59	49	15	29.72	6.72	49	15	29.84	6.55	49
Household head's age (years), descriptive only				16	42.69	13.61	98	16	43.21	13.87	95	19	43.43	13.86	95	18	43.27	14.23	98
Altitude (m)				2	828.59	648.21	2,626	5	994.27	586.62	2,600	6	960.93	590.12	2,602	0	952.49	592.21	2,323
Precipitation				36.26	99.42	33.77	225.16	43.62	113.58	28.91	252.70	43.14	104.59	23.46	211.71	38.72	89.25	32.90	391.08
Maximum temperature				19.00	28.86	2.22	33.20	18.67	27.89	2.47	33.17	20.57	28.83	2.20	33.57	14.33	27.51	2.43	31.95
Minimum temperature				9.00	19.06	3.32	24.40	8.33	18.06	3.23	24.33	8.43	18.77	2.96	24.57	7.00	17.63	3.43	23.83

“Missing/not recorded” categories reflect data recording defect; roof material in particular is missing for the majority of the sample after 2007/08. Percentages are unweighted; applying DHS sample weights changes the overall estimates only marginally (e.g., anemia prevalence 59.8% weighted vs. 60.1% unweighted; malaria positivity 12.6 vs. 10.8%) but does not alter the patterns.

Maternal characteristics were relatively stable across the survey years. The mean age of mothers, linked directly to each child's own birth-history record, was 29.73 years (SD = 6.62). A fifth of mothers (20.99%) had no formal education, over half (52.59%) had primary education, and 13.19% had secondary education or above; the remaining 13.23% of children were not living with an identifiable biological mother at interview and are reported separately rather than folded into any education category. Household composition showed that half of families (50.2%) consisted of 6–10 members, while 35.2% had between 1 and 5 members. Socioeconomic status varied across households. The poorest and poorer wealth quintiles accounted for 21.8% and 20.8% of households, respectively. The remaining households were distributed across the middle (20.8%), richer (20.7%), and richest (16.0%) quintiles. Sanitation and housing conditions reflected the predominantly rural context. Over half of the households surveyed (55.3%) were using unimproved toilet facilities. Water source was recorded for all but 0.5% of households, of which 70.0% relied on an unimproved source. Wall material was recorded for 79.1% of households, of which 69.3% had durable walls (bricks, blocks, or stone) and 9.7% traditional materials (grass, mud, or wood); the remaining 20.9% were not recorded, concentrated in the 2011/12–2022 rounds. Roof material was recorded for only 35.3% of households overall (and as few as 3.7% in 2015/16); among those with a recorded value, 22.3% had grass/thatch/mud roofing and 13.0% had more durable coverings such as iron sheets, tiles, or concrete. Because roof material is missing for the majority of the sample in every round after 2007/08, it should be interpreted with caution and is not comparable to the near-complete water, wall, and toilet variables.

The spatio-temporal distribution of anemia and malaria among children under 5 years in Tanzania across the four surveyed years shows contrasting patterns of occurrence over time (Figure 1). Anemia remains widespread across the country throughout the study period, with red circles indicating clusters where anemia was present and blue circles indicating its absence (Figure 1A). The distribution shows little apparent reduction in anemia occurrence over time, suggesting that factors beyond malaria may contribute to its persistent burden. In contrast, malaria occurrence exhibits a marked decline over the study period, with red circles representing clusters where malaria was present and blue circles representing those without malaria (Figure 1B). Fewer malaria-positive clusters are observed in 2015/16 and 2022 compared with the earlier survey years, indicating a substantial reduction in the spatial occurrence of malaria over time.

**Figure 1:**
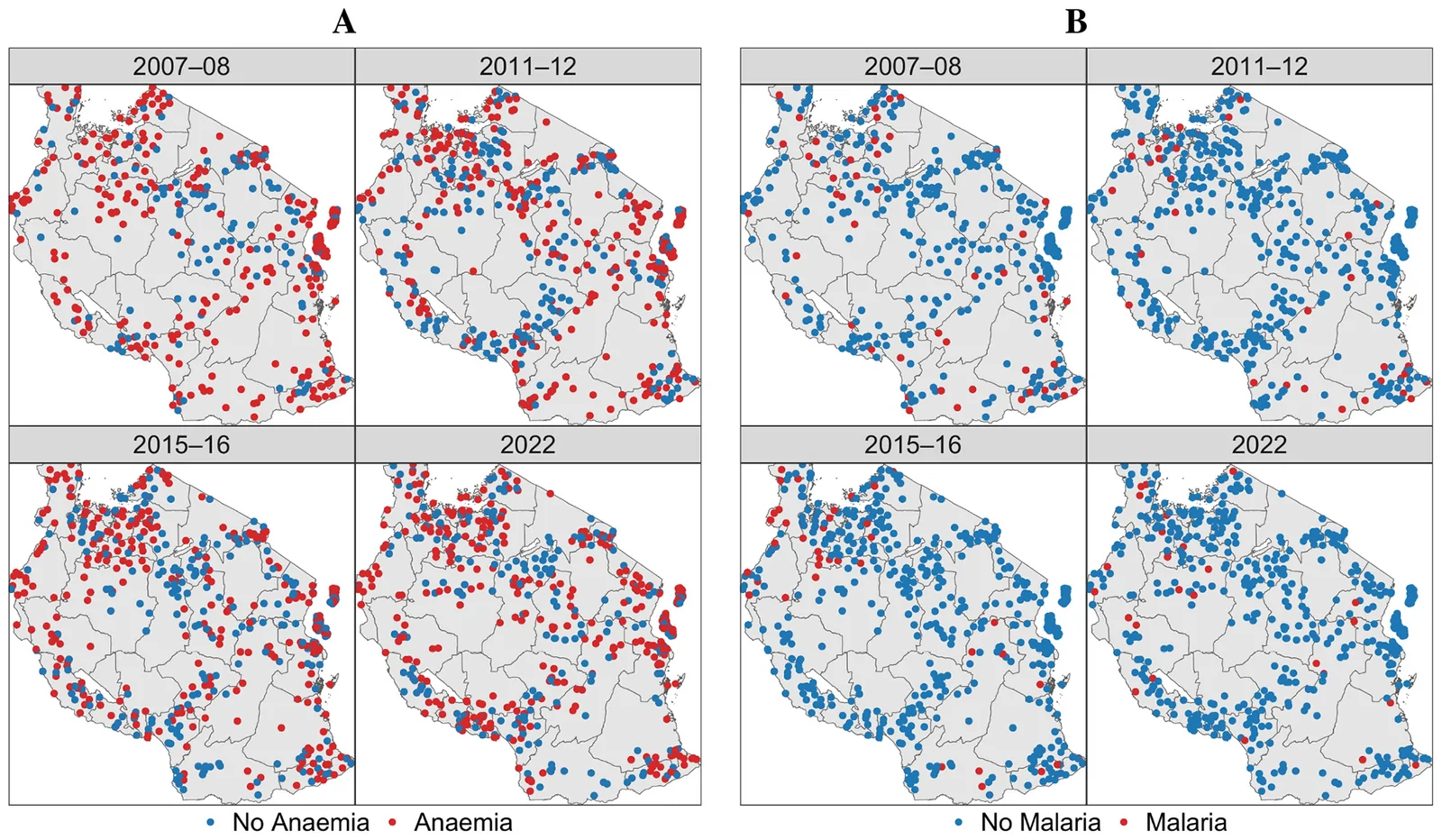
Spatial and temporal distribution of anemia and malaria in Tanzania for children under 5 years of age across the survey years 2007/08, 2011/12, 2015/16 and 2022. Each circle represents a sampled and georeferenced cluster. **(A)** Anemia occurrence. **(B)** Malaria occurrence.

### 3.2 Model fit and validation

To justify the spatio-temporal model structure and to evaluate out-of-sample predictive performance, we compared the primary spatio-temporal specification, an SPDE spatial field combined with an AR(1) temporal structure, against two alternatives: a spatial-only model [SPDE field without AR(1)] and a non-spatial model (fixed effects with RW2 smooth terms only). Model fit was assessed using the deviance information criterion (DIC) and the Watanabe–Akaike information criterion (WAIC; results reported in Supplementary Table S5). The spatio-temporal model was preferred over the non-spatial specification (ΔDIC ≈236, ΔWAIC ≈196). A static spatial field without temporal structure performed no better than omitting spatial correlation entirely, indicating that the time-varying AR(1) component, rather than spatial correlation alone, was responsible for the improvement in fit. We additionally tested the random-effects structure and smooth-term specification against alternative specifications. Finally, given that malaria was the covariate of primary substantive interest, we tested whether its association with anemia varied across three plausible effect-modifying dimensions: child's age group, household wealth quintile, and urban/rural residence. The urban/rural interaction did not improve fit and its interaction term did not exclude the null. Both the age-group and wealth-quintile interactions produced small improvements in DIC and WAIC relative to the no-interaction model (age: ΔDIC ≈−6, ΔWAIC ≈−3; wealth: ΔDIC ≈−9, ΔWAIC ≈−4). We therefore based the choice of which interaction to include in the final model on the pattern of its stratum-specific terms rather than on the aggregate fit statistics alone: for child's age group, the oldest stratum (42–59 months) showed a credible interval excluding the null, and the ratio of malaria odds ratios increased monotonically and biologically coherently across the three age strata; for household wealth, only one of four strata (“poorer”) excluded the null, in a non-monotonic pattern that mirrors the same boundary-level uncertainty already noted for the wealth main effect, consistent with sampling variation rather than a genuine dose-response interaction. We therefore included only the child's age-group interaction in the final model.

We evaluated the predictive performance of the model using spatially blocked five-fold cross-validation, withholding entire geographic blocks of clusters rather than individual observations. These assessments included the mean absolute error, root mean squared error, and calibration of predicted probabilities for out-of-sample (Supplementary Table S6). The model achieved an area under the ROC curve of 0.700 and an 11.5% improvement in Brier score relative to a no-skill baseline. Predicted probabilities were well-calibrated in nine of ten deciles, with the model modestly under-predicting risk in the lowest decile (predicted mean 0.31 vs. observed 0.34). Additionally, 93.3% of held-out cluster-rounds fell within their 95% binomial prediction interval.

#### 3.2.1 Anemia indicators in children under 5 years of age

Malaria status emerged as a strong predictor of anemia, with children who had malaria showing substantially higher odds of being anemic than those without malaria (OR = 3.53; 95% CrI: 3.18–3.92), providing strong evidence that malaria infection substantially increases the likelihood of anemia (Figure 2A). Male children exhibited a 19% higher likelihood of anemia compared with female children (OR = 1.19; 95% CrI: 1.13–1.25). Although children living in urban areas exhibited a slightly higher odds of anemia compared with their rural counterparts, the credible interval crossed one (OR = 1.10; 95% CrI: 0.99–1.22), indicating no statistically significant difference in anemia risk by place of residence after adjusting for other covariates. Children whose mothers achieved primary education were 16% less likely to be anemic (OR = 0.84; 95% CrI: 0.78–0.91), while those whose mothers completed secondary or higher education were 23% less likely (OR = 0.77; 95% CrI: 0.68–0.87), relative to children of mothers without formal education. Children who were not living with an identifiable biological mother were also somewhat less likely to be anemic than those whose mother had no formal education (OR = 0.85; 95% CrI: 0.77–0.95), plausibly reflecting older caregiver and child age composition in this group. A similar pattern was observed with household wealth. Children from households classified in at least the “middle” wealth quintile were significantly less likely to have anemia compared with those from the poorest households, with a progressive reduction in anemia risk as household wealth increased; the “poorer” quintile also marginally excluded the null (*OR* = 0.92; 95% CrI: 0.84–1.00), though this interval sits at the boundary and should be interpreted cautiously rather than as a firm effect distinct from the poorest group. Household size was also associated with anemia risk: children from households with 6–10 members were 7% more likely to be anemic (OR = 1.07; 95% CrI: 1.01–1.15), and children from large households (more than 10 members) were 23% more likely to be anemic compared with those from households with fewer than five members (OR = 1.23; 95% CrI: 1.12–1.35). None of the “missing/not recorded” indicator terms (toilet, water, wall, roof) reached credible significance, indicating that the extensive missingness identified is not acting as a hidden confounder in the model.

**Figure 2:**
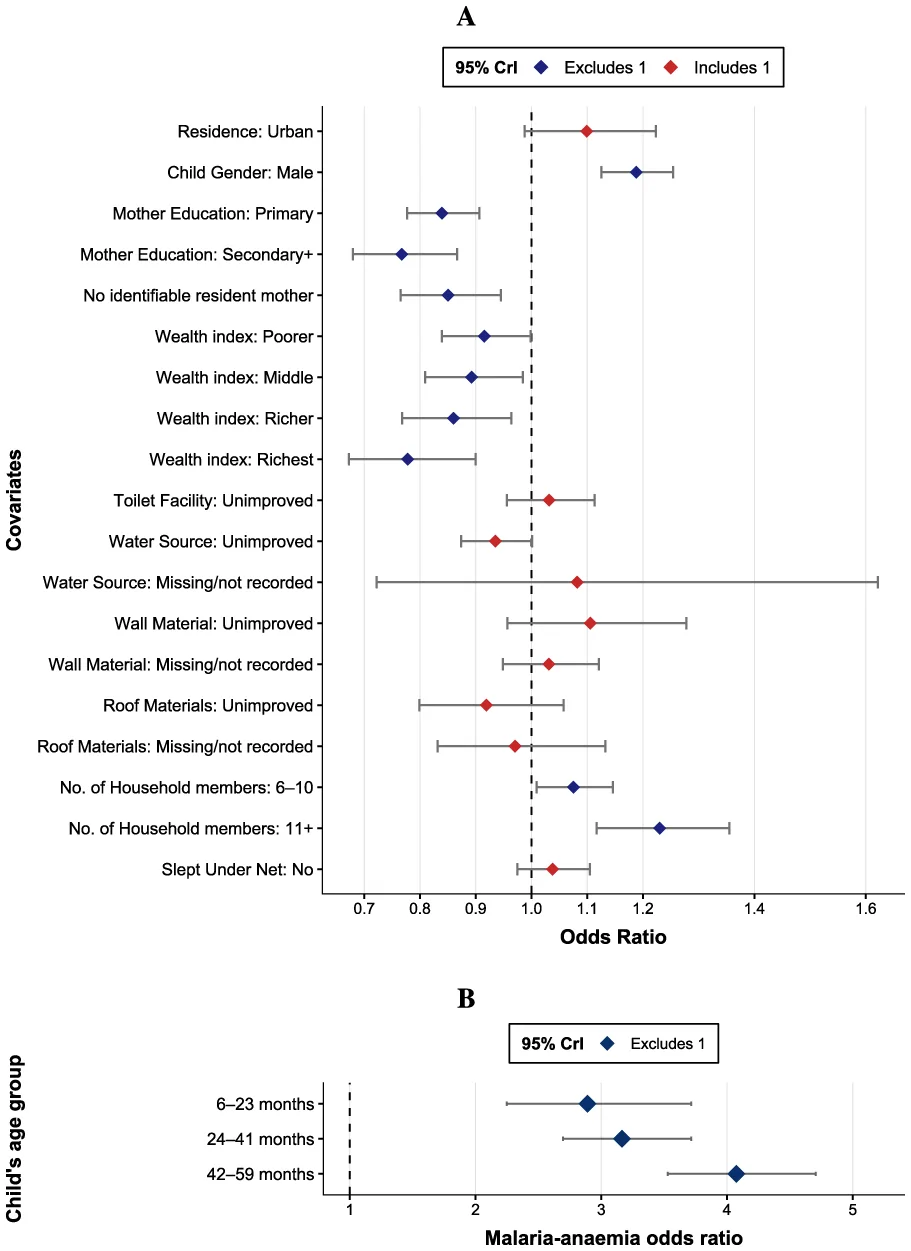
**(A)** Estimated odds ratio and their 95% credible intervals for all-cause of anemia. **(B)** Malaria-anemia odds ratio by child's age stratum, from the age×malaria interaction term: the odds ratio rises monotonically from 2.89 (95% CrI: 2.25–3.72) among children 6–23 months to 3.17 (2.70–3.72) at 24–41 months and 4.08 (3.53–4.71) at 42–59 months.

The malaria-anemia odds ratio increased monotonically with age, from 2.89 (95% CrI: 2.25–3.72) among children 6–23 months to 3.17 (2.70–3.72) at 24–41 months and 4.08 (3.53–4.71) at 42–59 months (oldest vs. youngest ratio of odds ratios 1.41, 95% CrI: 1.06–1.87 (Figure 2B)). This gradient is biologically coherent: anemia in the youngest children is driven by multiple concurrent factors (iron stores at birth, weaning-related dietary transitions), against which malaria's marginal contribution is proportionally smaller, whereas by 42–59 months other drivers of anemia have receded and malaria constitutes a larger share of the remaining risk. Additional results are available in Supplementary Table S7.

The estimated non-linear associations between key environmental variables and the log-odds of childhood anemia show distinct patterns across the observed ranges of the covariates (Figure 3). Each panel presents the posterior mean effect (red line) together with its 95% credible interval (black lines). The estimated effect of precipitation is essentially flat and close to zero across the full observed range, with the credible interval encompassing zero throughout, suggesting little evidence of an independent association between precipitation and anemia after accounting for other covariates, including malaria (Figure 3A). This pattern is consistent with the possibility that the relationship between precipitation and anemia operates primarily through malaria transmission rather than through an independent residual effect of rainfall. The smooth effect of maximum temperature also provides limited evidence of an independent association, as the credible interval includes zero across the entire observed range (Figure 3B). Although the posterior mean is positive at the coolest temperatures, declines through zero at approximately 25°C, and becomes increasingly negative above 30°C, the credible interval widens substantially at higher temperatures where cluster density is sparse. Overall, this pattern is consistent with a weak independent effect of maximum temperature after adjustment for other covariates, including malaria. In contrast, minimum temperature exhibits a more pronounced and monotonic association with anemia (Figure 3C). Values below approximately 15°C are associated with a credibly reduction in the log-odds of anemia, with the effect crossing the null over a transition zone of approximately 15–17°C and becoming credibly positive from approximately 18°C onward, increasing steadily toward the upper end of the observed range.

**Figure 3:**
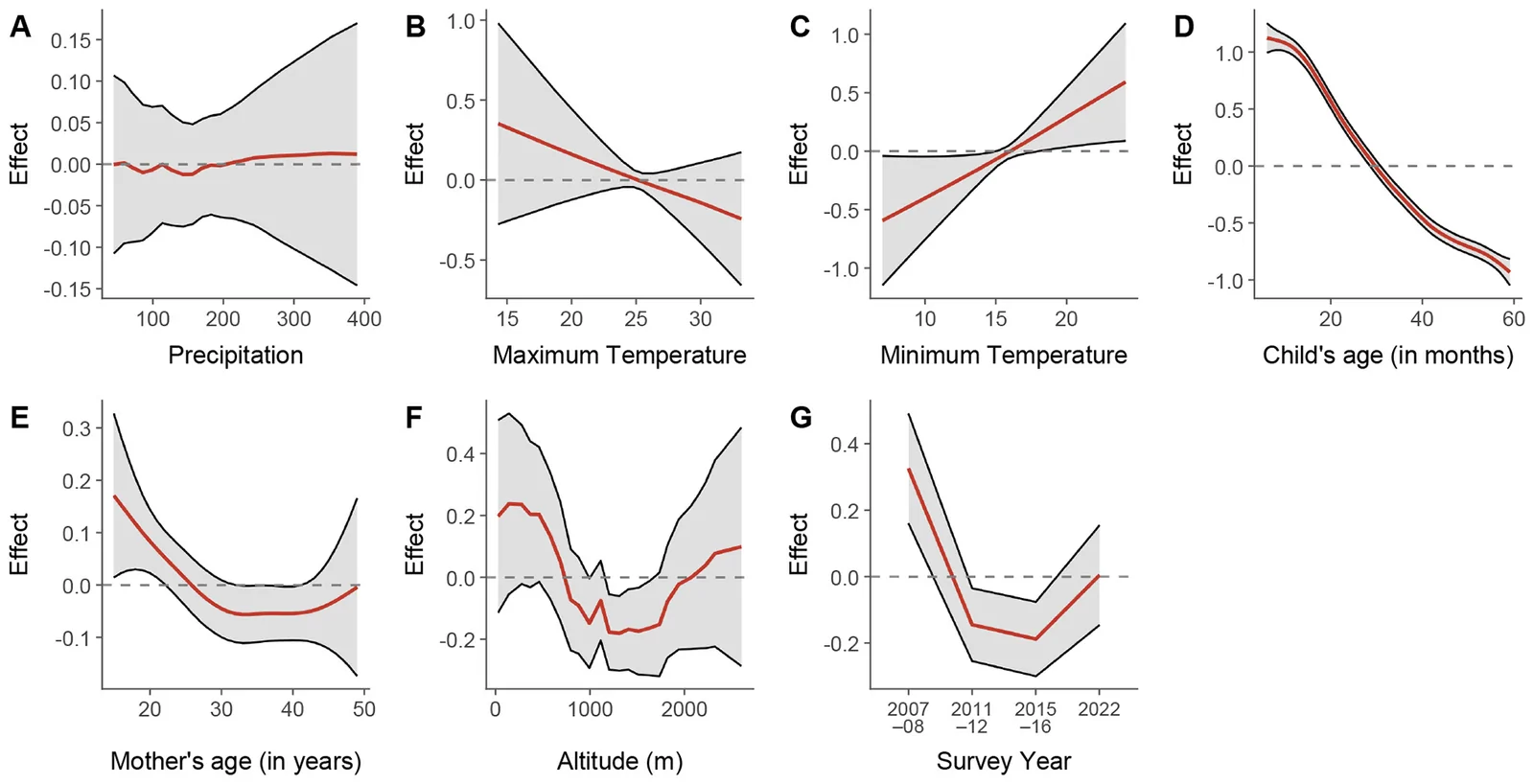
Non-linear effects of environmental variables [precipitation **(A)**, maximum temperature **(B)**, minimum temperature **(C)**], child's age **(D)**, mother's age **(E)**, cluster altitude **(F)**, and the non-linear temporal trend **(G)** on childhood anemia in Tanzania. The red lines represent the posterior mean of each smooth effect, and the black lines show the 95% credible intervals. **(B–E, G)** Are modeled using a second-order random walk (RW2) prior; **(A, F)** use a first-order random walk (RW1) prior, found to be better supported for these two terms in a model-specification robustness check.

The non-linear effects of child age and maternal age likewise show distinct patterns in relation to the log-odds of childhood anemia (Figure 3). The log-odds of anemia decrease monotonically with increasing child age, indicating that younger children have a higher risk of anemia (Figure 3D). Maternal age shows a clear gradient, with the log-odds of anemia being highest and most uncertain among the youngest mothers, particularly around 15 years, before declining steadily through the twenties (Figure 3E). The estimated effect reaches a plateau of credibly reduced log-odds between approximately 33 and 40 years, after which the credible interval widens at the oldest maternal ages, around 49 years, and again encompasses zero. Cluster altitude shows a non-linear association with anemia, with the estimated effect being imprecise and compatible with zero below approximately 1,000 m and above roughly 1,800 m, where cluster density is sparse (Figure 3F). Across the intermediate altitude range of approximately 1,000–1,800 m, the effect is credibly negative, indicating lower log-odds of anemia at these elevations. This pattern is compatible with reduced malaria transmission at higher elevations within this intermediate range, although the altitude effect should be interpreted in the context of the other covariates included in the model. Finally, the non-linear temporal trend shows a notable decline in the log-odds of childhood anemia between the 2007/08 and 2015/16 survey rounds, followed by an increase in 2022 (Figure 3G).

#### 3.2.2 Predicted anemia prevalence

The spatial predictions show the progression of childhood anemia prevalence across Tanzania over the four survey years, 2007/08, 2011/12, 2015/16, and 2022 (Figure 4). Each column presents the predicted mean prevalence together with the lower (0.025 quantile) and upper (0.975 quantile) credible limits obtained from the Bayesian spatio-temporal geostatistical model. In 2007/08, the country showed widespread high predicted anemia prevalence, with particularly increased levels across the western regions, the Lake Zone, the southern areas, and parts of the southern highlands. The upper credible limits suggest that some areas may have experienced even higher risks, while the lower credible limit still indicates a broad distribution of moderate to high prevalence across much of the country. By 2011/12, the spatial distribution of childhood anemia prevalence remained largely unchanged, with only modest indications of improvement. Slight reductions were evident in parts of the western regions and the southern highlands; however, the overall burden persisted at substantial levels and continued to be widespread across the country. Moving into the period between 2011/12 and 2015/16, the maps reveal only limited progress. Although a few areas experienced modest declines in prevalence, many regions, particularly in the southern and Lake Zone areas, continued to exhibit high predicted values. By 2022, the spatial distribution of anemia prevalence reveals a renewed increase, with more areas in the southern, western, and Lake Zone regions displaying increased predicted mean prevalence. The upper credible limits reinforce this pattern, showing both greater uncertainty and consistently higher potential risk in these regions. This re-emergence of higher prevalence highlights a concerning reversal and highlights the need for strengthened, targeted interventions to address childhood anemia across multiple regions of Tanzania.

**Figure 4:**
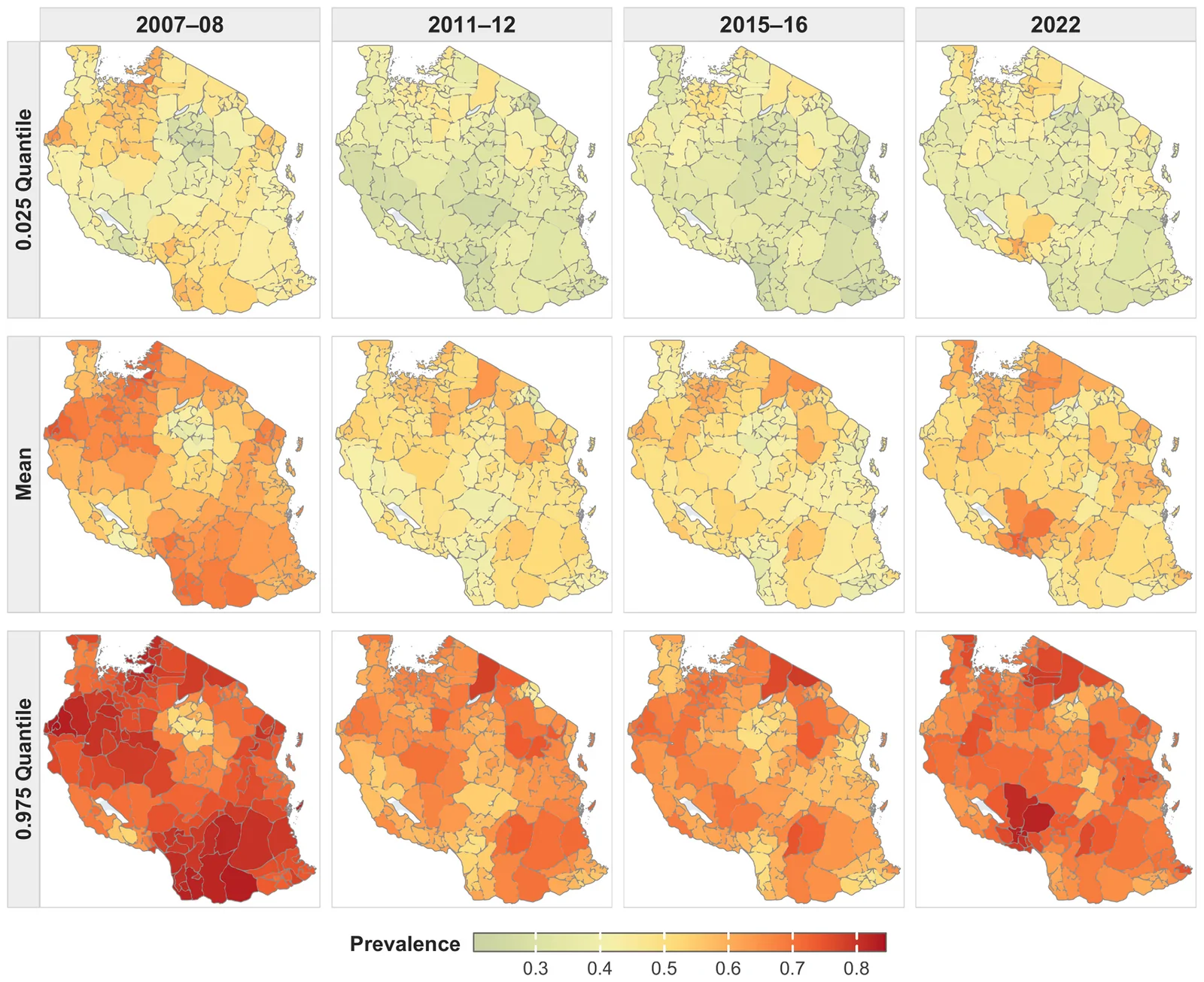
Predicted mean prevalence of childhood anemia across Tanzania for each survey year, shown alongside the corresponding lower (0.025 quantile) and upper (0.975 quantile) credible limits from the Bayesian spatio-temporal geostatistical model. Predictions from the continuous spatial surface are averaged within district boundaries (195 districts) for display; a small number of districts with no surface grid point falling inside them (0.8% of national land area) are assigned the value of the nearest grid point.

The posterior standard deviations of the model-based predictions illustrate the spatial and temporal distribution of uncertainty in childhood anemia prevalence across Tanzania (Figure 5). The maps quantify the uncertainty associated with the predicted prevalence at each location and survey period, based on the spatio-temporal Stochastic Partial Differential Equation (SPDE) model with a first-order autoregressive (AR(1)) temporal structure. Uncertainty is higher in regions with sparse survey coverage, particularly in the central, western, and southern Tanzania, where predictions depend more heavily on spatial interpolation and temporal smoothing. In contrast, coastal, eastern, lake zone, and southern highland areas with dense DHS cluster data exhibit consistently low uncertainty, reflecting strong model support and stable spatial patterns. Across survey years, uncertainty generally declines from 2007/08 to 2015/16, indicating improved data availability and effective temporal borrowing within the model. However, a notable increase is observed in 2022. Nevertheless, the magnitude of uncertainty remains modest, suggesting that the model provides reliable and well-constrained predictions for most regions of the country.

**Figure 5:**
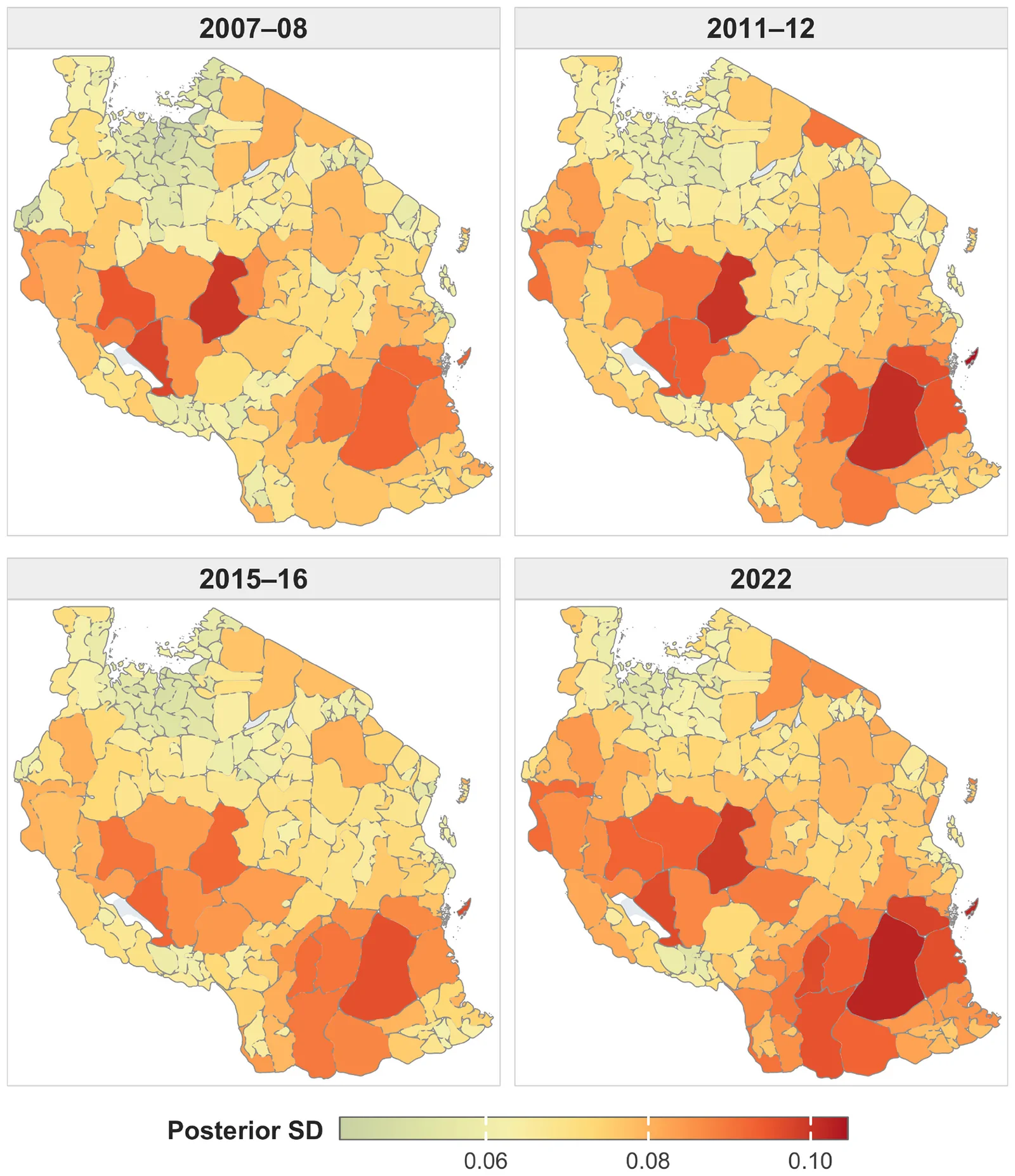
Posterior standard deviation of model based predictions of anemia prevalence across Tanzania for each survey year. Averaged within district boundaries (see Figure 4).

### 3.3 Causal analysis of malaria on childhood anemia

The regression estimates for malaria, obtained from year-stratified logistic regression models regressing childhood anemia on malaria status while adjusting for the full set of observed covariates, were 0.845, 0.896, 1.301, and 1.241 for the survey years 2007/08, 2011/12, 2015/16, and 2022, respectively (Table 3). All coefficients were statistically significant (*p* < 0.0001), indicating a strong and consistent positive association between malaria and childhood anemia across survey periods. The resulting ACE estimates (Equation 5), were 0.155, 0.197, 0.266, and 0.246 for 2007/08, 2011/12, 2015/16, and 2022, respectively (Table 3). Under the identification assumptions stated in the Methods (conditional exchangeability, positivity, consistency, and temporality), these represent the expected population-level increase in the probability of anemia attributable to malaria, after standardizing for the observed covariates; because several of these assumptions cannot be fully verified from the available data, the estimates are best read as evidence consistent with a causal effect rather than confirmation of one. Positive ACE values across all survey years are consistent with malaria acting as a determinant of childhood anemia in Tanzania, conditional on those assumptions holding. Specifically, the ACE estimate of 0.266 in 2015/16 indicates that malaria exposure was associated with an average absolute increase of approximately 26.6 percentage points in the probability of childhood anemia during that period, representing the year when malaria exerted its strongest causal influence. The ACE increased from 0.155 in 2007/08 to 0.197 in 2011/12, peaked at 0.266 in 2015/16, and declined to 0.246 in 2022, suggesting a persistent yet time-varying causal relationship between malaria and childhood anemia.

**Table 3:** Malaria effect estimates from the model regressing childhood anemia on malaria prevalence, adjusted for other covariates, with the corresponding average causal effects (ACE).

Survey year	Malaria estimates (anemia regressed on malaria)			Average causal effects (ACE)		
	Estimates	Standard error	*p*-value	Estimates	Standard error	95% CI
2007/08	0.845	0.109	< 0.0001	0.155	0.012	(0.132–0.180)
2011/12	0.896	0.109	< 0.0001	0.197	0.020	(0.160–0.235)
2015/16	1.301	0.098	< 0.0001	0.266	0.016	(0.232–0.297)
2022	1.241	0.185	< 0.0001	0.246	0.028	(0.187–0.299)

Fitted with design-based survey-weighted regression, with 95% intervals from a stratified cluster bootstrap.

To further quantify the impact of malaria on childhood anemia, we estimated the Attributable Fraction (AF) (Equation 3) and the Number Needed to Treat (NNT) (Equation 4) across the four survey years: 2007/08, 2011/12, 2015/16, and 2022 (Table 4). These metrics provide insight into both the population level burden of malaria on anemia and the individual level effectiveness of malaria interventions, and consistently indicate that malaria substantially contributes to anemia prevalence among children under 5 years of age in Tanzania. The AF was 3.85% (95% CI: 3.22–4.47) in 2007/08, suggesting that approximately 4% of anemia cases in children in Tanzania could have been prevented in the absence of malaria. The AF declined slightly to 3.27% (95% CI: 2.45–4.14) in 2011/12 and peaked at 6.37% (95% CI: 5.23–7.43) in 2015/16, indicating that malaria's contribution to anemia was highest during this period. By 2022, the AF declined to 3.22% (95% CI: 2.32–4.22), yet remained non-negligible, highlighting the continued relevance of malaria as a contributing factor to anemia.

**Table 4:** Attributable fraction (AF) and number needed to treat (NNT) for malaria's impact on childhood anemia across survey years.

Surveyed year	2007/08	2011/12	2015/16	2022
AF (%) (95% CI)	3.85 (3.22, 4.47)	3.27 (2.45, 4.14)	6.37 (5.23, 7.43)	3.22 (2.32, 4.22)
NNT (95% CI)	6.68 (5.74, 7.90)	5.11 (4.27, 6.34)	3.93 (3.48, 4.50)	4.13 (3.38, 5.46)

Fitted with design-based survey-weighted regression with stratified cluster-bootstrap intervals.

The NNT estimates reinforce these findings by quantifying the efficiency of malaria prevention in reducing anemia. In 2007/08, the NNT was 6.68 (95% CI: 5.74–7.90), indicating that approximately seven children would need to be protected from malaria to prevent one case of anemia. This value decreased over time, reflecting improved intervention efficiency: 5.11 (95% CI: 4.27–6.34) in 2011/12, 3.93 (95% CI: 3.48–4.50) in 2015/16, and 4.13 (95% CI: 3.38–5.46) in 2022. The lowest NNT observed in 2015/16 coincides with the peak AF, suggesting that malaria interventions during this period were particularly impactful in reducing anemia burden. Although the NNT increased slightly in 2022, it remained lower than in earlier years, indicating sustained effectiveness of malaria control efforts.

## 4 Discussion

Childhood anemia remains a persistent and geographically public health concern in Tanzania, driven by a complex interplay of infectious, socioeconomic, and environmental determinants. Using a Bayesian spatio-temporal geostatistical framework applied to four rounds of nationally representative survey data, this study mapped the spatial and temporal distribution of anemia among children under 5 years, identified its key determinants, and estimated the causal impact of malaria under a set of identification assumptions. Despite gradual improvement over the past two decades, childhood anemia remained prevalent in Tanzania, affecting more than half of children under 5 years of age. National prevalence declined from 68.8% in 2007/08 to a low of 57.4% in 2015/16, before rising again to 59.7% in 2022. Throughout the study period, prevalence remained above the WHO threshold of ≥40% that defines a severe public health problem in this age group (1, 34). This persistent high prevalence suggests that existing interventions have had limited impact and highlights the need for multisectoral strategies that address both nutritional deficiencies and infectious causes. The patterns observed in this study are consistent with trends reported across sub-Saharan African countries, where anemia continues to pose a major health challenge among children under 5 years. Studies from Ghana, Kenya, Nigeria, and Uganda have reported prevalence rates above 50%, with malaria, nutritional deficiencies, and socioeconomic deprivation identified as the primary determinants (35–38).

The strong association between malaria and childhood anemia, coupled with the substantial ACE and AF estimates, supports the biological and epidemiological evidence that malaria is one of the major cause of anemia in endemic settings (39–41). *Plasmodium falciparum*, which is responsible for the large majority of malaria infections in Tanzania, causes haemolysis, suppresses erythropoiesis, and triggers inflammatory responses that impair red blood cell production, collectively contributing to a rapid decline in hemoglobin concentrations in young children (42, 43). This aligns with other studies which show that anemia is more common in children with malaria; in a single-site cohort of Kenyan children, *Plasmodium falciparum* was estimated to account for nearly half of severe anemia cases, although the generalisability of this estimate to other settings, including Tanzania, has not been established (44, 45). The observed temporal variation, particularly the peak malaria-attributable fraction in 2015/16, aligns with national malaria trends and likely reflects fluctuating transmission intensities influenced by vector ecology, intervention coverage, and climate variability (9, 46). The malaria-anemia odds ratio also strengthened with child age, from 2.89 among children 6–23 months to 4.08 among those 42–59 months. This is consistent with the youngest children's anemia risk being driven by several concurrent, largely malaria-independent factors, such as depleting neonatal iron stores and the dietary transition at weaning, against which malaria's marginal contribution is proportionally smaller; by 42–59 months, once these early-life drivers have receded, malaria accounts for a comparatively larger share of the remaining risk.

The finding that maternal education is protective against anemia is supported by other studies showing that higher maternal education is associated with improved nutrition practices, better hygiene, and higher use of preventive services, all of which contribute to reduced anemia burden (47, 48). Lower maternal education and household poverty significantly increased the risk of anemia (49), consistent with evidence across low- and middle-income countries linking anemia to inadequate dietary diversity, limited access to health services, and delayed care-seeking (50, 51). Although sanitation-related variables (toilet facility, water source, wall and roof materials, and ITN use) did not show statistically credible independent associations in the multivariable model, their effects may be mediated through other covariates such as wealth and malaria status. Poverty limits access to iron-rich foods, clean water, sanitation, and mosquito-control measures, and these pathways are likely captured collectively by the wealth index. The higher risk of anemia in larger households may reflect increased exposure to infections and competition for nutritional resources, affecting child development (52), consistent with previous research showing that household composition is an important yet under-addressed risk factor (53). Children of the youngest mothers (around 15 years) had the highest and most uncertain anemia risk, risk declined through the twenties, and reached a plateau of significantly reduced risk between approximately 33 and 40 years. This is consistent with evidence that very young, often first-time, mothers may have less caregiving experience and greater nutritional vulnerability themselves, both of which can translate into poorer child anemia outcomes. Children who were not living with an identifiable biological mother at interview were, somewhat unexpectedly, less likely to be anemic than children of mothers with no formal education. We interpret this cautiously: this group is disproportionately in older, more often female-headed (frequently grandparent-led) households, and may over-represent slightly older children whose iron stores and immune status are more developed; it should not be read as evidence that maternal absence is protective.

The non-linear climatic effects observed in this study are broadly consistent with established pathways linking climate to malaria transmission and, in turn, to anemia risk, although the strength of evidence differs by variable. Higher precipitation is well-established in the entomological literature as expanding mosquito breeding habitats and increasing malaria transmission (54–56); however, in our data the direct, residual association between precipitation and anemia was essentially null once appropriately regularized, which is consistent with precipitation acting on anemia risk indirectly, through malaria (already included as a direct covariate in the model), rather than through an independent pathway detectable in the smooth term itself. Temperature patterns also align with entomological evidence: moderately warm temperatures enhance mosquito survival and parasite development, while excessively high temperatures suppress transmission (57, 58). Multi-country analyses show that a 1 °C rise in annual temperature increases the odds of childhood anemia by approximately 14% (59), and region-specific studies across 43 African countries demonstrate a heterogeneous and non-linear relationship between temperature and anemia (60).

The predicted anemia prevalence maps revealed marked geographic heterogeneity, with the highest burden concentrated in the western regions, Lake Zone, southern areas, and parts of the southern highlands. These spatial patterns align with the known distribution of malaria transmission in Tanzania (12, 61), and are consistent with regional analyses across sub-Saharan Africa showing that malaria hotspots and anemia hotspots spatially overlap (34, 62, 63). The co-occurrence of high malaria and anemia burden in the same zones highlights the importance of spatially targeted integrated interventions, particularly in areas where malaria control remains suboptimal. The limited spatial change between 2007/08 and 2015/16 suggests that while malaria control and nutrition interventions have likely contributed to some reductions, persistent transmission and heterogeneity have continued to sustain anemia in many high-risk areas. The resurgence in predicted anemia prevalence in 2022 may also mirror disruptions in essential health and nutrition services during the COVID-19 pandemic, which have been shown to reverse gains in child health outcomes (64, 65). Our findings highlight the importance of geographically targeted, integrated strategies that jointly address malaria control, nutrition-related factors, and health system strengthening to effectively reduce childhood anemia in Tanzania and similar endemic settings.

This study had several strengths. The Bayesian spatio-temporal SPDE framework, implemented in R–INLA with survey design weights, accommodated complex spatial and temporal dependence, allowed for non-linear covariate effects, and generated high-resolution prevalence surfaces with quantified uncertainty. Integrating causal inference extended the analysis beyond conventional associations and provided population-level effect estimates that are directly interpretable for intervention planning. The inclusion of four nationally representative survey rounds spanning 15 years enabled the assessment of long-term temporal trends that cannot be captured in single cross-sectional studies.

Despite these strengths, several limitations warrant consideration. The underlying data are cross-sectional, and although causal modeling was applied, residual unmeasured confounding cannot be fully excluded. The causal analysis relied on year stratified logistic regression rather than the spatio-temporal model because counterfactual prediction requires integrating over the latent spatial field, a computation that is infeasible at this scale. Consequently, the ACE, AF, and NNT estimates do not incorporate spatial heterogeneity in malaria effects and may therefore represent population averages that obscure important geographic variation. Anemia was defined solely by hemoglobin concentration, without distinguishing among its biological causes, such as iron deficiency, folate deficiency, helminth infections, or other micronutrient deficiencies, which vary geographically and may confound the malaria–anemia relationship.

A further limitation concerns the adjustment set, which omits several plausible shared causes of both malaria exposure and anemia. These include dietary iron and micronutrient intake, supplementation status, deworming history, sickle cell trait, HIV status, recent febrile illness or antimalarial use, and breastfeeding practices. Most of these variables are not collected in DHS surveys. Given the biological plausibility of these pathways, their omission represents a substantive threat to the conditional exchangeability assumption rather than a routine residual limitation. Malaria status was determined using rapid diagnostic tests (RDTs), which detect parasite antigens that may persist for several weeks after parasite clearance. Some RDT-positive children may therefore not have been parasitaemic at the time of hemoglobin measurement, a misclassification that would tend to attenuate rather than inflate the estimated malaria–anemia association. Microscopy data were not available in these survey rounds, preventing a sensitivity analysis comparing RDT-based and microscopy-based case definitions. Finally, DHS cluster GPS coordinates are randomly displaced (jittered) by up to 2 km in urban areas, 5 km in rural areas, and 10 km for a random 1% of rural clusters to preserve respondent confidentiality. This positional displacement introduces noise into the extraction of climate covariates and attenuates the estimated spatial range. Because the displacement is on the same order as, or larger than, parts of the 50 km minimum mesh resolution used here, it may modestly smooth or blur fine-scale spatial structure in the predicted prevalence surfaces.

## 5 Conclusion

Childhood anemia in Tanzania remains a major public health concern, characterized by persistently high prevalence, marked spatial heterogeneity, and strong influence from malaria, socioeconomic factors, and climatic factors. This study's causal analysis, under the stated assumptions, is consistent with malaria continuing to exert a significant and time-varying impact on anemia among children under 5 years, with the average causal effect peaking at 0.266 and the attributable fraction reaching 6.37% in 2015/16. The NNT declined from 6.68 in 2007/08 to 3.93 in 2015/16, indicating that malaria prevention efforts during this period were particularly efficient in reducing the anemia burden. The findings also highlight the critical roles of maternal age, maternal education, household wealth, household size, and climatic conditions in shaping the risk of anemia, while sanitation and housing variables did not show independent credible associations after adjusting for other covariates. Spatial predictions revealed persistent high-risk areas in the western, southern, and Lake Zone regions, highlighting the need for geographically targeted, multisectoral interventions that integrate malaria control with nutritional and socioeconomic support.

## Data Availability

Publicly available datasets were analyzed in this study. This data can be found here: https://www.dhsprogram.com/.
