# Supplementary material for: Bayesian spatio-temporal modeling of childhood anemia in Tanzania: spatial distribution, determinants, and causal impact of malaria
Source: Front Public Health. 2026 Oct 21;14:1919182. doi: 10.3389/fpubh.2026.1919182 (PMC13746951; doi:10.3389/fpubh.2026.1919182)
Supplement: Supplementary file 1 [file Supplementary_file_1.pdf]

Supplementary Material

1 SUPPLEMENTARY TABLES AND FIGURES

1.1 Tables

Table S1. Supplementary Table S2. Model comparison across nested spatio-temporal specifications, reporting the deviance information criterion (DIC) and Watanabe-Akaike information criterion (WAIC); lower values indicate better fit.

**Model specification**	**DIC**	**WAIC**
Non-spatial (fixed + smooth effects only)	31648.9	31901.0
Spatial-only (SPDE field, no AR(1) temporal structure)	31533.6	31800.7
Spatio-temporal (SPDE field with AR(1), model without the age interaction)	31400.6	31693.6
Spatio-temporal (SPDE field with AR(1)) + malaria $\times$ child's age group (final model)	**31394.8**	**31690.2**

Table S2. Supplementary Table S3. Spatially-blocked 5-fold cross-validation of the primary spatio-temporal model. Clusters, rather than individual children, were assigned to folds so that spatially proximate clusters remain together, giving an honest assessment of out-of-sample predictive performance in unsampled areas.

**Metric**	**Value**
Mean absolute error (MAE) of predicted probability	0.423
Root mean squared error (RMSE) of predicted probability	0.461
Area under the ROC curve (AUC)	0.700
Brier skill score vs. no-skill baseline	11.5%
Cluster-round coverage of 95% prediction interval (nominal 95%)	93.3%

Table S3. Supplementary Table S1. Odds ratios with 95% credible intervals for factors associated with anaemia among children under five years in Tanzania from the survey years 2007/08, 2011/12, 2015/16, and 2022. Estimates are from the final model, which includes the malaria × child's age-group interaction; the malaria row gives the age-averaged odds ratio from the same model without the interaction.

**Covariates**	**OR (95% CrI)**
Presence of malaria	
No	1
Yes, age-averaged	3.534 (3.184, 3.924)
Place of residence	
Rural	1
Urban	1.099 (0.988, 1.223)
Gender	
Female	1
Male	1.188 (1.125, 1.254)
Mother's highest education	
None	1
Primary	0.839 (0.777, 0.907)
Secondary school or above	0.767 (0.679, 0.867)
No identifiable resident mother	0.850 (0.765, 0.945)
Family wealth quintile	
Poorest	1
Poorer	0.915 (0.839, 0.998)
Middle	0.893 (0.809, 0.984)
Richer	0.860 (0.768, 0.964)
Richest	0.778 (0.672, 0.900)
Toilet facility	
Improved	1
Unimproved	1.032 (0.956, 1.113)
Water source	
Improved	1
Unimproved	0.935 (0.874, 1.001)
Missing/not recorded	1.082 (0.722, 1.622)
Wall exterior main material	
Improved (bricks/blocks/stone)	1
Unimproved (grass/mud/wood)	1.106 (0.957, 1.278)
Missing/not recorded	1.031 (0.949, 1.121)
Roof main material	
Improved (iron sheets/tiles/concrete)	1
Unimproved (grass/thatch/mud)	0.919 (0.799, 1.058)
Missing/not recorded	0.971 (0.832, 1.133)
Number of household members	
1–5	1
6–10	1.075 (1.009, 1.146)
11 or more	1.230 (1.117, 1.355)
Slept under net	
Yes	1
No	1.038 (0.975, 1.105)
**Malaria × child's age-group interaction**	
Malaria-anaemia odds ratio, by age stratum	
6–23 months (reference)	2.890 (2.247, 3.716)
24–41 months	3.165 (2.696, 3.714)
42–59 months	4.075 (3.529, 4.705)
Ratio of malaria odds ratios vs. 6–23 months reference	
24–41 vs. 6–23 months	1.095 (0.818, 1.466)
42–59 vs. 6–23 months	1.410 (1.062, 1.872)
**Random effects / Hyperparameters**	**Mean (95% CrI)**
Survey clusters, Precision ($u_{c(i)}$)	17.39 (11.47, 25.92)
Sampling strata, Precision ($v_{s(i)}$)	48.44 (17.97, 114.47)
Spatio-temporal field (ξ)	
Spatial range, $\hat{\rho}$ (km)	165.1 (116.5, 227.0)
Spatial SD, $\hat{\sigma}_{\varphi}$	0.499 (0.426, 0.582)
AR(1) temporal correlation, $\hat{a}$	0.439 (0.212, 0.635)
